# Conceptual expansion of photomedicine for spatiotemporal treatment methods

**DOI:** 10.1039/d4md01005a

**Published:** 2025-03-11

**Authors:** P. K. Hashim, Ashwin T. Shaji, Ammathnadu S. Amrutha, Shifa Ahmad

**Affiliations:** a Research Institute for Electronic Science, Hokkaido University Kita20, Nishi 10, Kita-ku Sapporo Hokkaido 001-0020 Japan hashim@es.hokudai.ac.jp; b Graduate School of Life Science, Hokkaido University Kita 10, Nishi 8, Kita-ku Sapporo Hokkaido 060-0810 Japan; c School of Chemistry, Indian Institute of Science Education and Research Thiruvananthapuram Kerala 695551 India

## Abstract

Photomedicine has evolved from basic phototherapy to a broad range of light-based technologies to achieve precise and minimally invasive therapeutic outcomes. Recent advances in light sources, photochemical reactions, and photoswitches have facilitated the development of light-activated methodologies for modulating biological processes. This review discusses the history of light therapy that leads to the emergence of a new field known as photopharmacology, mode of actions in photopharmacology such as photodynamic, photo-uncaging and photoswitchable methods, a few representative examples in photopharmacology, and a brief overview of its associated challenges. The current developments in photopharmacology hold great promise for the treatment of diseases such as cancer, with enhanced therapeutic precision, and minimal side effects. We foresee further expansion of photomedicine for novel approaches in precision medicine and healthcare, and unprecedented treatment methods.

## Abstract 2

This review covers the history of light therapy, the emergence of photopharmacology, its methods, and challenges. Current advancements show promise for treating diseases like cancer with precision and minimal side effects.

## Graphical abstract

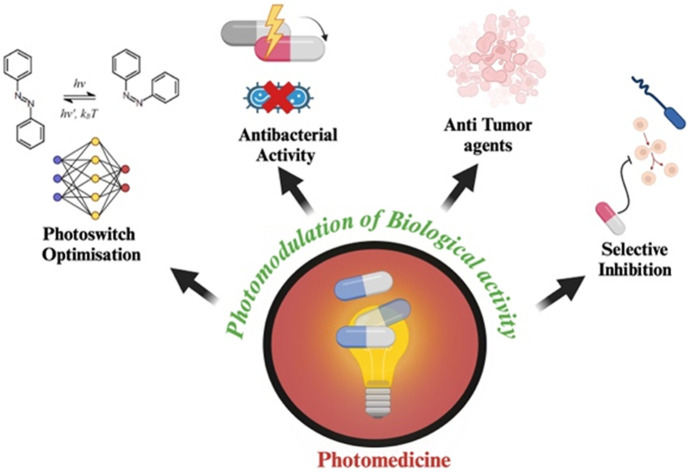

## 1. Introduction

Over 85% of small-molecule drugs in clinical development are discarded due to their poor drug selectivity, despite having potential to treat currently untreatable diseases.^1^ Poor drug selectivity arises when a drug binds to unintended targets, reducing its therapeutic efficacy and increasing the likelihood of side effects. A typical example of poor drug selectivity is the severe side effects, such as hair loss, seen with chemotherapy for cancer. Additionally, drug resistance, particularly with antibiotics, arises from overuse and accumulation in the body, leading to the selection of resistant bacterial strains and reduced treatment effectiveness.^2^ The core issue of poor selectivity and side-effect is the uncontrolled activity of drugs in time and space, when and where the therapeutic effect is not beneficial. This demands the need for innovative molecular approaches that allow dynamic control over drug activity, enabling selective action only when and where it is beneficial. Light presents unique advantages as a non-invasive tool for regulating biological systems. Unlike chemical agents, which can interfere with biological processes, photons are orthogonal to most chemical and biochemical components, ensuring low toxicity. Additionally, light can be delivered with exceptional spatial and temporal accuracy, which is required for fine-tuning biological responses.

Photomedicine is a branch of medicine that uses light to diagnose, treat, and monitor diseases. This field mainly encompasses light therapy and light-based technologies to achieve precise and minimally invasive therapeutic outcomes.^3*a*^ Photomedicine has undergone significant advancements over the years, evolving from basic phototherapy techniques to a broad spectrum of innovative methods. A notable progress is the birth of a new subfield known as photopharmacology, in which a molecular system having photosensitive and biologically active segments produces distinguishable biological response before or after light irradiation. In this review, we categorize photopharmacology as a specialized branch within photomedicine focusing on photoswitchable bioactive compounds. We discuss the history of light therapy and the emergence of photopharmacology, mode of actions and working principles, developments in the field of photopharmacology, a few representative examples, and associated challenges.

### 1.1 Brief history of photomedicine

**Fig. 1:**
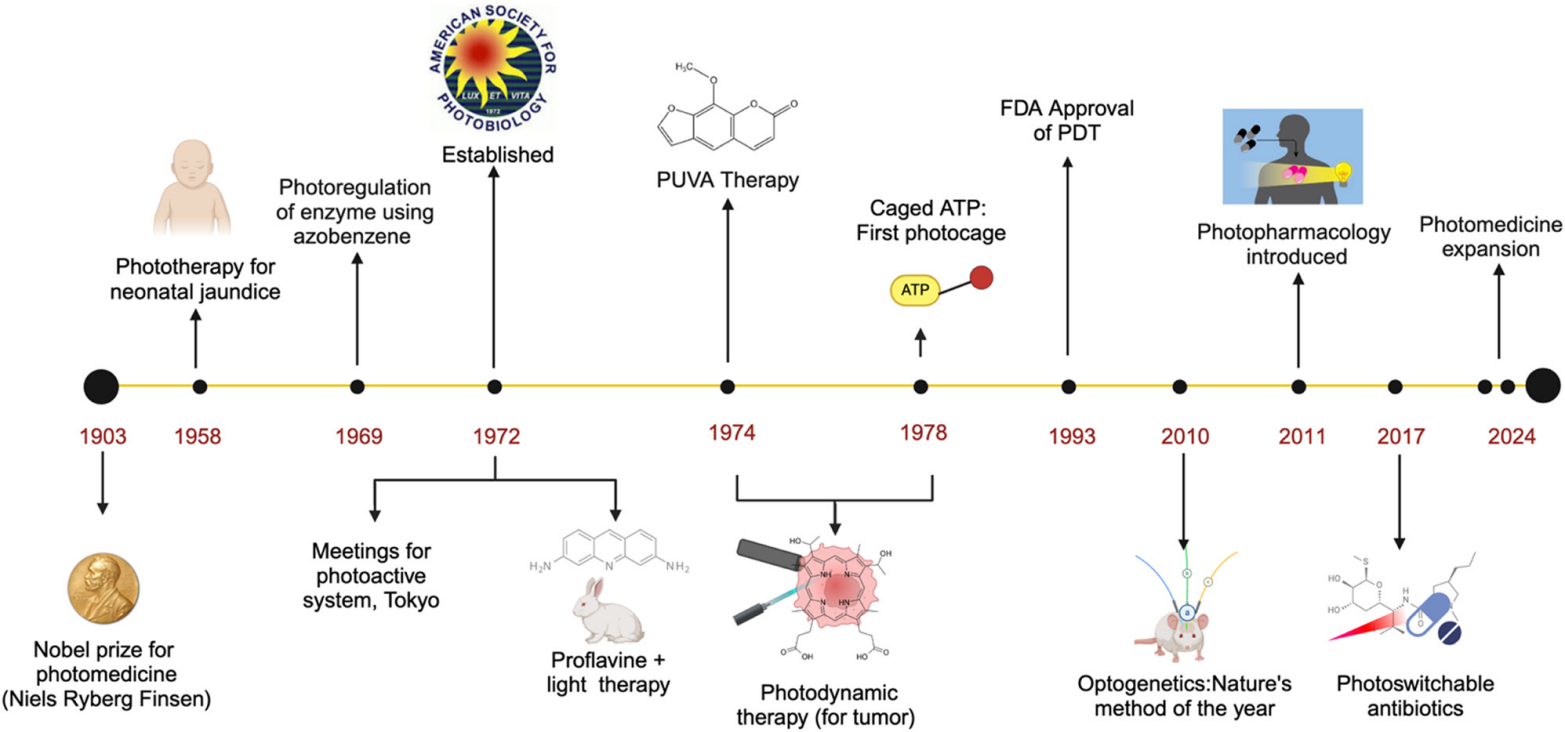
Timeline showing the sequence of events that led from the onset of photomedicine to modern-day photopharmacology.

In a broad sense, the history of photomedicine dates back to ancient Egyptian medical practices. For instance, heliotherapy (sunlight treatment) has been used for centuries to treat skin diseases (*e.g.*, vitiligo) by using a boiled extract of the *Ammi majus* plant followed by sun exposure. The Atharva-Veda in India was also described using *Psoralea corylifolia* seeds and sunlight for treating “leucoderma”.^3*b*^ The main idea behind phototherapy is that chromophores in the skin (such as DNA, amino acids, pigments, and small molecules) absorb light, triggering photochemical reactions that lead to photobiological responses. For effective therapy, focused light is preferred over scattered sunlight, and many specialized light sources were developed. Modern phototherapy began with Niels Ryberg Finsen, who is considered the father of ultraviolet therapy. In 1896, Finsen developed a “chemical rays” lamp to treat “lupus vulgaris”, successfully curing his friend's disease. He later created the “Finsen Lamp”, a focusable carbon-arc torch, with which he treated over 800 lupus patients with 80% cure rate. At a time when antibiotics and anti-inflammatory drugs were unavailable, Finsen's work was groundbreaking. In 1903, he was awarded the Nobel Prize in Medicine for his contributions to treating “lupus vulgaris” with concentrated light rays, the only Nobel Prize ever given for dermatology or photomedicine.^3*c*^ With the discovery of broadband ultraviolet B (UVB) in 1925 and Osram Ultravitalux lamps in 1960, the phototherapy method has gained much attention for treating several diseases.^4^ One of the earliest uses of phototherapy was for treating neonatal jaundice in 1958. In this treatment, light energy alters the structure of bilirubin, converting it into molecules that can be more easily excreted from the body, helping to reduce jaundice in newborns.^5,6^ In an effort to formalize the field of photomedicine, key international meetings were also held in Tokyo (1972) and other countries, as well as the establishment of the American Society for Photobiology (1972) (Fig. 1).^7,8^

Another important study was reported in 1972 that used proflavine in combination with light from a 150 watt incandescent lamp to treat “herpes keratitis” in rabbits.^9^ Although the herpetic keratitis could be rapidly treated by the proflavine-light combination, concerns were raised over potential carcinogenic effects, as tumor formation was observed by detailed investigations.^10,11^ Around the same time, researchers also started to focus on the isolation of plant compounds for using it in therapeutic application in combination with light. A notable method was developed in 1974 known as psoralen and ultraviolet A (PUVA) therapy, which combined the 8-methoxypsoralen (8-MOP) isolated from *Ammi majus* with UVA radiation to treat psoriasis.^12,13^ The effectiveness of PUVA therapy was confirmed by clinical trials involving thousands of patients.^14^ A variation of this treatment, known as bath PUVA, involves psoralen baths followed by UVA exposure and is still in use today because it helps minimize the side effects of 8-MOP, such as nausea and dizziness.^15^ To increase the efficacy of PUVA therapy, new high-intensity ultraviolet light sources such as narrowband UVB (311–313 nm) and later UVA1 (340–400 nm) and UVA2 (320–340 nm) were also developed.^16,17^

The use of chemical dyes as photosensitizers, basic principles behind the discovery of “photodynamic therapy”, began in the early 19th century, following a serendipitous discovery by Raab *et al.* They observed that irradiation of paramecia (single-celled organisms) with visible light, in the presence of a dye, resulted in the death of the organisms.^18,19^ It took several decades to show the clinical use of the photosensitization method until important clinical research in which malignant tumors were significantly reduced by the combined action of a hematoporphyrin derivative and red light.^20^ Continued work revealed the basis for the oxygen- and light-dependent photodynamic reaction which is now known as photodynamic therapy (PDT).^21,22^ In 1994, PDT using porfimer sodium and an excimer dye laser (P-PDT) was approved by the Japan Ministry of Health and Welfare for early-stage lung, esophageal, gastric, and cervical cancers. Nowadays, PDT is a treatment method on the commercial scale for esophageal cell carcinoma.^23^

**Fig. 2:**
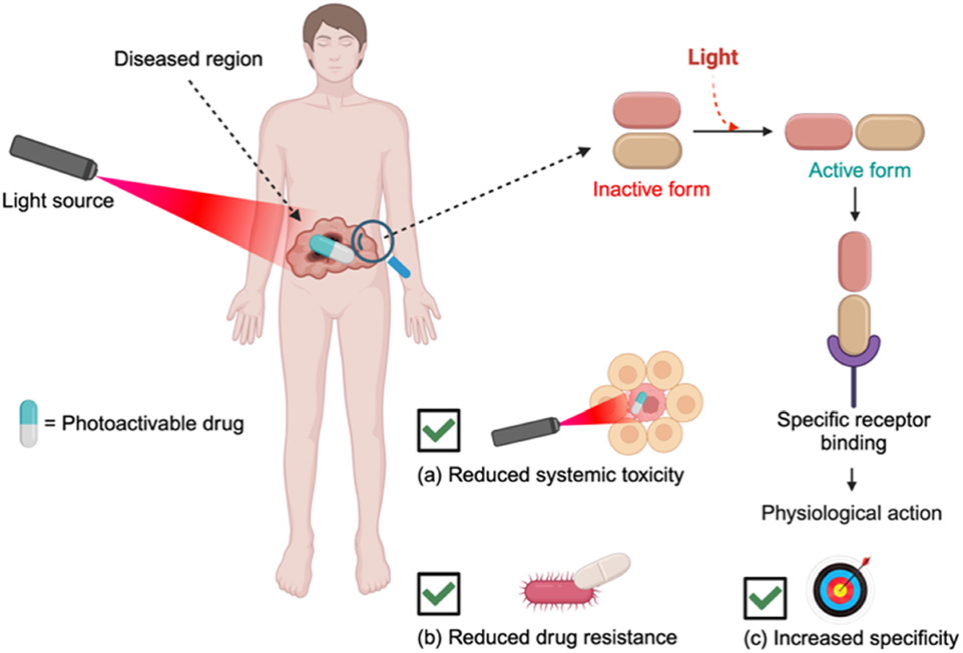
Simplified scheme highlighting the key features of photopharmacology such as (a) reduced drug toxicity, (b) reduced drug resistance and (c) enhanced precision by limiting the drug activation at target sites.

Another type of photomedicine is the structural change of inactive molecule by light irradiation to an active form for certain biological response. There was an early report of this type in 1969 by the groups of Erlanger and Nachmansohn, who showed the reversible photoregulation of azobenzene-based inhibitors of acetylcholinesterase.^24,25^ In 1977, J. J. Voorhees and W. Wierenga patented an approach, where the methotrexate drug was inactivated by photochemically labile blocking groups and activated therapeutic effects only upon light exposure at the target site (U.S. Patent Serial No. 787320). Another related category was a “caged ATP” developed by Hoffman *et al.* in 1978, where the photolabile group attached to adenosine 5′-triphosphate can be cleaved by light irradiation.^26^ These early studies paved the way for today's advanced light-activated methods, generally known as “photopharmacology”, which use light stimuli and innovative photo-active molecular designs to precisely control the biological activity in space and time. Unlike conventional drugs, photopharmacological drugs remain inactive until exposed to a specific wavelength of light, allowing for exact spatial and temporal regulation of drug activation (Fig. 2). This light-dependent activation significantly reduces systemic side effects, as drugs can be triggered only at targeted sites, enhancing treatment precision, and minimizing unintended damage to healthy tissues.

## 2. The biological window

**Fig. 3:**
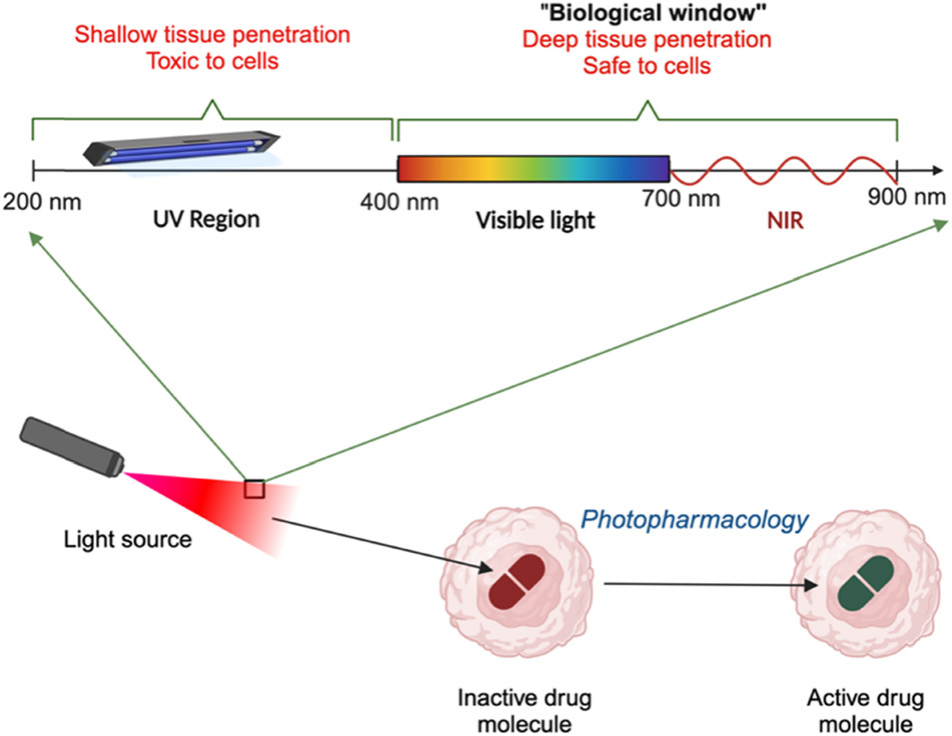
Scheme showing the range of light wavelengths used in photomedicine, highlighting the ideal biological window across the visible and near-infrared (NIR) regions.

In photomedicine, the selection of light is crucial, as it determines both the safety and effectiveness of treatment. Light of different wavelengths interacts uniquely with biological tissues. High-energy UV light is limited by low tissue penetration and toxic to the biological cells.^27–29^ The visible (400–700 nm) and near-infrared (NIR, 700–900 nm) light region arguably known as the ‘biological window’, as the light can penetrate tissues efficiently and hence is the best choice for biological applications (Fig. 3). Although visible light is generally safe, shorter-wavelength visible light has limited penetration in blood-rich tissues due to absorption by biomolecules like hemoglobin. However, recent advances in engineering and materials science lead to the development of wireless subdermal electronics, multimodal fiber optics, and light-conversion materials, which can deliver light directly to the target site.^30–33^ By positioning the irradiation source near the tissue of interest, it is now possible to partly overcome the light penetration issues in photomedicine applications.

## 3. Modes of action

Photopharmacology encompasses three major modes of action: photodynamic therapy (PDT), photo-uncaging, and photoswitching. PDT is at a commercial level whereas photocaging and photoswitching are still at the academic research level and are not yet translated to the clinic. Optogenetics is another method that relies on light for dynamically controlling biological functions especially brain and neural.^34^ Optogenetics was recognized as “Method of the Year 2010” by ‘Nature’ for its capability to control neuronal activity using genetically encoded, light-sensitive proteins. Readers may find excellent review articles related to the field of optogenetics.^35,36^

### 3.1 Photodynamic therapy (PDT)

**Fig. 4:**
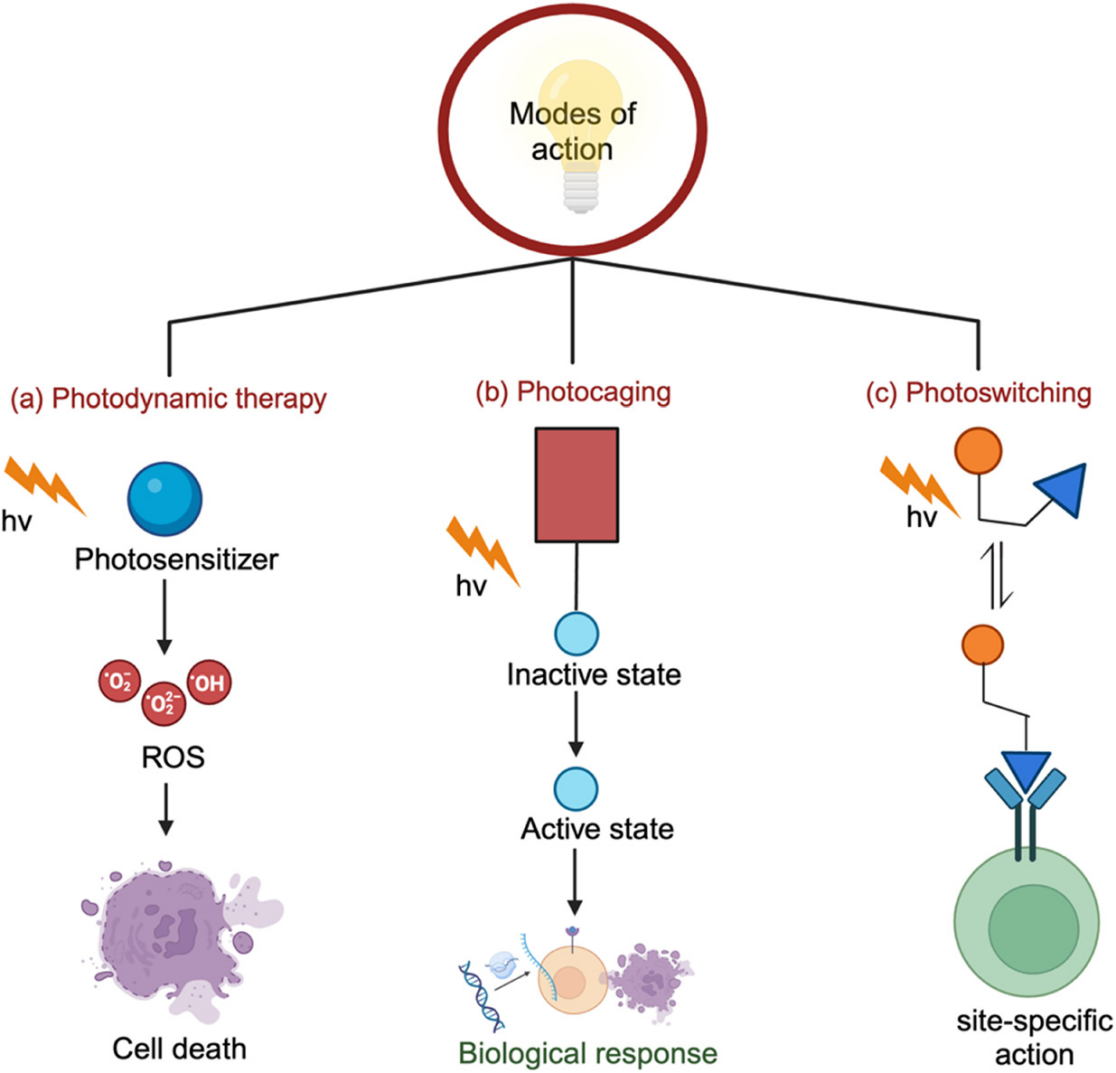
Scheme illustrating the three modes of action in photopharmacology: a) photodynamic therapy, b) photo-uncaging, and c) photoswitching.

In this mode of action, a light-activated drug (also known as photosensitizers in PDT) administered to a patient remains inactive up until exposure to a specific wavelength of light. The activation of the photosensitizer by light leads to the generation of reactive oxygen species (ROS), which causes cellular damage by disrupting cellular components such as cell membranes, and ultimately leading to cell death (Fig. 4a). The mechanism of PDT was reviewed previously.^37^ Briefly, it involves two main pathways: type I and type II. In the type I mechanism, the excited photosensitizer produces reactive radicals and ions (hydroxyl radicals, superoxide anions), which cause cellular damage by interacting with lipids, proteins, and DNA. The type II mechanism primarily generates singlet oxygen, a highly reactive species that induces lipid peroxidation and damage to cellular components. While the type I mechanism leads to a variety of reactive species, the type II pathway focuses on the destructive power of singlet oxygen. Both mechanisms contribute to the therapeutic effects of PDT, and their balance depends on factors like the photosensitizer, tissue type, and light conditions. The photosensitizers are of various types and the first family discovered was hematoporphyrin (Hp) derivatives, which are now available as commercial products (*e.g.*, Photofrin®, Photosan, Photocan).^38,39^ Chlorines, a chlorophyll like substance and purins, degradation products of chlorophyll, and phthalocyanine dyes also showed excellent photosensitizing properties including some success in the clinical trials.^40^ Readers may refer to several excellent reviews for recent progress in the development of photosensitizers.^41^ Following the earlier studies reported for skin cancer treatment by PDT,^42^ various types of cancers, and infections, are also investigated in clinical PDT.^43–46^

### 3.2 Photo-uncaging

In this mode of action, therapeutic activity of the original drug is masked by photolabile protecting groups (PPGs) forming caged drugs. When irradiated with light of a suitable wavelength, these caged drugs undergo a photocleavage reaction, releasing the active drug (Fig. 4b). For instance, anticancer drugs such as imatinib and vemurafenib can be conjugated with PPGs (*e.g.*, 4,5-dimethoxy-2-nitrobenzyl or *o*-nitrobenzylic) to create caged drugs, which undergo a cleavage reaction by 365 nm-UV light.^47,48^ The PPGs based on the nitrobenzyl platform is not ideal for clinical use due to potential tissue damage and limited penetration depth associated with UV exposure. Alternate caging groups that are responsive to longer wavelengths in the visible or near-infrared (NIR) range are also reported. One such approach utilized a cyanine dye nanocage carrying gabapentin, a therapeutic agent for traumatic brain injury (TBI). When exposed to NIR light, the cyanine nanocage triggered the selective release of gabapentin at the TBI-affected site.^49^ Another strategy to shift the photoactivation wavelength into the longer visible or NIR ranges is the use of bulky aromatic groups, such as xanthenium, coumarin, BODIPY and cyanine.^50–53^ However, the release of bulky leaving groups by photoreaction can lead to undesirable side-effects. In contrast, a caging technique recently developed by Tamaoki *et al.* introduced a less-bulky dimethylamino leaving group at the core imidazole structure of a triarylimidazole-based anticancer drug. This caged system showed visible-light-triggered photo-uncaging, and subsequent release of the active drug for the biological effect demonstrated in a wound-healing assay.^54^

A related category of photo-uncaging is by using an inorganic complex, which relies on the photochemical bond cleavage of either a metal–ligand coordination bond or a carbon–oxygen bond.^55,56^ As most of the studies in this category have focused on cancers, this subfield of photo-uncaging is also known as photoactivated chemotherapy (PACT). In PACT treatment of cancer, the prodrug is poorly toxic in its photocaged form, while very toxic to cancer cells after light activation. Many metal centers, especially ruthenium metal (Ru), have been reported as efficient photocaged compounds that are activated with visible or NIR light.^57,58^ Though many clinical trials are attempted, Ru-based PACT is still in its infancy as it is not yet applied in patients.

### 3.3 Photoswitching

This mode of action includes a photoswitch segment that can reversibly change its conformation upon light exposure, switching the drug between active and inactive forms (Fig. 4c).^59–63^ Photoswitches are isomerized by light irradiation resulting in two structurally different forms *i.e.* the *trans* and *cis* isomers whose properties (polarity and stability) are different. Azobenzene is a traditional photoswitch that undergo *trans*–*cis* isomerization by UV light.^64^ Examples of other photoswitches which undergo photoisomerization include diarylethenes, spiropyrans, indigoids, fulgides, *etc.*^65,66^ Despite the limitation of UV irradiation, initial studies were conducted using azobenzene as the most commonly used chromophore for controlling the photoswitch-incorporated biological action.^64^ Then, researchers developed visible light/NIR active azobenzene derivatives by structural modifications through *ortho* and *para* substitution to the azobenzene core.^67^ Another category of photoswitches is based on “azoheteroarenes”, which have attracted significant interest due to their distinct photophysical properties originating from the five-membered heteroaryl groups.^68^ Examples of commonly used five-membered heteroaryl motifs in photoswitch design include pyrrole, pyrazole, imidazole, isoxazole, triazole, thiazole and thiophene. These types of photoswitches showed very different optical properties, steric arrangements, and molecular shapes than the azobenzene derivatives.^69–71^ Since many drugs contain the five-membered heteroaryl groups, their photoswitchable derivatives can be effectively utilized in photopharmacology, provided they undergo isomerization by longer wavelength visible light.^72–74^

As the majority of the examples in photopharmacology use photoswitches, their design criteria are important to discuss. Readers may refer to an excellent review focusing on the rational design in photopharmacology with molecular photoswitches.^75^ Briefly, there are three approaches for the design of photoswitches, traditional molecular design strategies, quantum calculations, and machine learning (ML). Each of these methods has advantages and disadvantages and one can select the design optimization methods based on the specific need. Traditional molecular design is based on principles from organic chemistry, photophysics, and molecular engineering, which select a molecular core (*e.g.*, aryl azo) that undergoes reversible photoisomerization upon light absorption.^76^ To achieve light absorption in the visible light spectrum (400–700 nm), the molecular core is modified by altering its conjugation length or introducing electron-donating (*e.g.*, OMe, NH_2_, NMe_2_) or electron-withdrawing (*e.g.*, halogens, CN, NO_2_) groups. However, to validate the expected photophysical properties, organic synthesis is the only method that sometimes causes tedious experimental work. Quantum calculations provide near accurate information of photophysical properties by taking the electronic structure and quantum energy levels of the photoswitch molecules.^77^ Density functional theory (DFT) and time-dependent DFT (TD-DFT) are the commonly used methods to calculate absorption spectra, geometry optimization of different isomeric states (*e.g.*, *cis* and *trans*), activation energy barriers, relaxation pathways from excited states, and thermal reversion kinetics from less stable isomers. Though quantum calculations provide detailed insights into the photoswitch design, they are very time consuming and require specialized expertise in computational chemistry.

The ML method is relatively new in the field of designing photoswitches and only a handful of outcomes are reported. For the prediction of photoswitch properties such as absorption spectra, and thermal half-life, ML models (*e.g.*, random forests, support vector machines, and neural networks) can be trained on large datasets of known photoswitchable molecules. In the reported case, the trained models successfully predicted the absorption maxima of new molecules based on their chemical structures.^78^ In terms of structure optimization of new molecules with desired properties, generative models (*e.g.*, variational autoencoders and generative adversarial networks) can be applied. One great advantage of ML is the significantly reduced time required for photoswitch optimization compared to other methods. For instance, the development of adiabatic artificial neural networks (DANN) has accelerated the virtual screening of azobenzene derivatives with remarkable speed and accuracy, six orders of magnitude faster than conventional quantum chemistry methods.^79^ Another approach utilizing a multi output Gaussian process trained on multiple photoswitch absorption wavelengths outperforms TD-DFT in terms of computational efficiency.^80^ Another approach integrates DFT calculations with ML models that allow the mechanistic study of photoisomerization and new thermal pathways in donor–acceptor Stenhouse adduct photoswitches.^81^ A recent study has shown the combination of ML and DFT methods for the prediction of the thermal half-lives of azobenzene derivatives and their trade-offs with maximum absorption wavelengths.^82^ Although ML methods show significant potential for photoswitch design, reliable ML model construction requires raw datasets with photoswitch properties under identical conditions. However, such datasets are currently limited, as experimental studies often measure photophysical properties under varying conditions, including different temperatures and solvents. This variability complicates the creation of consistent and robust datasets needed for accurate ML model training.

## 4. Bio-applications of photopharmacology

The field of photopharmacology is still in its infancy but rapidly progressing with many unprecedented targets that show precise control of biological action in cell-free assay, cultured cells, or animal models. We highlight a few representative examples that show irreversible (photo-uncaging) or reversible (photoswitch) changes in biological responses by light irradiation.^83^

### 4.1 Photoswitchable antibiotics

**Fig. 5:**
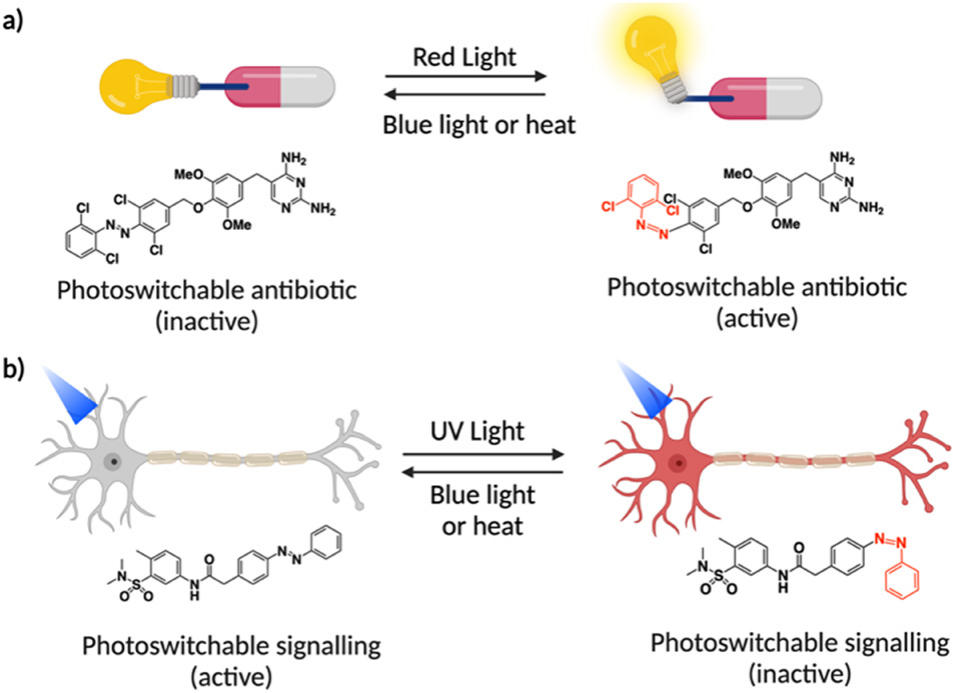
Scheme illustrating the concept of photoswitchable antibiotics (a) and signalling (b) along with the structural change of the azobenzene derivatives.

One way to address bacterial resistance is *via* novel molecular strategies that use externally triggered antibiotic activation, along with auto-inactivation mechanisms. Photoswitchable antibiotics allow reversible isomerization between two isomers using light and/or heat. If the light-activated isomer is active, it will auto-deactivate through sunlight or heat after being excreted from the body. This reduces the environmental accumulation of toxic antibiotics, helping to combat drug-resistant pathogens.^84^ The first report of this category was by Feringa *et al.* where they developed light-activated broad-spectrum antibiotics using a modified quinolone containing photoactive azobenzene moiety in quinolone sensitive *Escherichia coli*.^85^ The photoresponsive antibiotics inhibited bacterial growth in the *cis* form obtained after light irradiation while showed normal growth in the *trans* form. As the *cis* form of photoswitchable antibiotics reverts thermally to the inactive *trans* form, this system is an example of auto-inactivation. However, the study on the photocontrol of antimicrobial activity relied on cytotoxic UV light. To address this issue, the same group developed a new series of photoswitchable antibiotics based on a diaminopyrimidine core modified with an azobenzene photoswitch whose activity can be controlled by visible light, within a therapeutic window (Fig. 5a).^86^ Notably, one of the diaminopyrimidines exhibited an over 8-fold increase in antimicrobial activity after red light (652 nm) irradiation. In another recent study, photoswitchable arylazopyrazole based norfloxacin antibiotics showed a high degree of bidirectional photoisomerization by UV/visible light, relatively high *cis* half-life, and good fatigue resistance.^87^ The *cis* isomers exhibited greater antibacterial activity than norfloxacin against Gram-positive bacteria.

### 4.2 Cell signaling

G-protein coupled inwardly rectifying potassium channels (GIRKs) are vital for the body's inhibitory signaling pathways. When Gβγ subunits from GPCRs bind to the GIRK channel, they open, allowing potassium ions to flow out and making the inside of the cell more negative, and decrease the likelihood of action potentials. This system is also crucial for regulating cardiac output, movement coordination, and cognitive functions. However, precision control of GIRK channels is challenging. Trauner *et al.*, a pioneer in the field of optical control of cell signaling, reported a photoswitchable system named light operated GIRK-channel opener (LOGO), where a light-responsive agonist activates GIRK channels in the dark but deactivates by UV light irradiation (Fig. 5b).^88^ LOGO is the first light-controlled potassium channel opener specifically targeting the GIRK1 subunit. They also controlled neuronal firing in hippocampal neurons in the animal model, where the zebrafish larvae moved in a light-dependent fashion. They also developed a visible light switchable version of LOGO that activates GIRK channels in the dark but deactivates by green light (400–540 nm) irradiation.^89^ Protein kinases are one other category that regulate many cellular processes and signaling pathways. Szymanski and Feringa *et al.* reported a photoswitchable inhibitor for casein kinase 1δ (CK1δ), by conjugating an azobenzene moiety with an existing inhibitor (LH846) and showed reversible modulation of inhibitory action in an assay system.^90^

### 4.3 Tubulin inhibitor

**Fig. 6:**
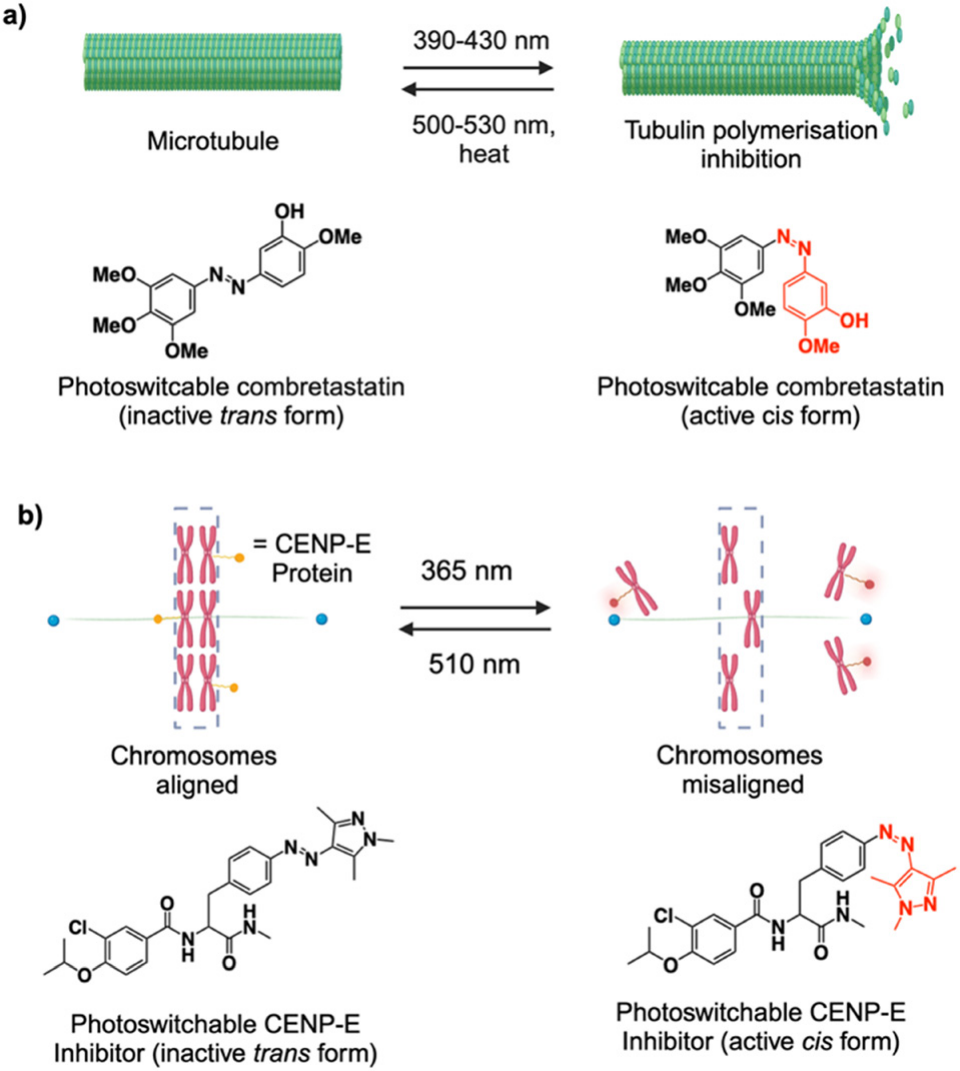
Scheme illustrating the concept of the photoswitchable tubulin inhibitor (a) and CENP inhibitor (b) along with the structural change of the azobenzene derivatives.

Tubulin-microtubule dynamics play an important role in cellular processes such as transportation, motility, and proliferation. In cancer therapeutics, small molecules that can interfere with the microtubule dynamics are one of the most clinically relevant categories of drugs. However, their inability to target specific cells leads to adverse side-effects. To address this issue, Borowiak, Trauner and Thorn-Seshold *et al.* developed photoswitchable tubulin inhibitors based on a combrestatin A-4 (CA4) core structure (named as photostatins), which can be switched on and off using visible light.^91^ In the *cis* form, the photostatins inhibit microtubule formation causing cytotoxicity, which was about 250 times higher as compared to the dark adopted *trans* form (Fig. 6a). They also showed modulation of microtubule dynamics in single live cells with sub-second response time and high spatial precision. Recently, to expand the scope of the photo-structure–activity-relationship, new azobenzene analogues of combrestatin were developed with tunable photoresponse wavelengths and lipophilicity.^92^ Other variants include photoswitchable paclitaxel-based microtubule stabilizers, wherein binding to the microtubule was induced by photoisomerization. They showed photo-control of microtubule dynamics with biological responses occurring on the order of seconds and spatial resolution at the single-cell level as well as individual neurites of primary neurons.^93^ Other categories for precise photomodulation of microtubule dynamics include pyrrole hemithioindigo- and colchicine-based inhibitors.^94,95^

### 4.4 Kinase inhibitors

Kinase inhibitors are an attractive class of compounds as some kinases are more active in cancer cells and blocking them may help to prevent cancer progression.^96^ However, the challenge is to achieve precise control over cell growth suppression avoiding severe side-effects. Centromere-associated protein E (CENP-E) facilitates the transport of chromosomes along spindle microtubules from the spindle poles toward the equatorial plate, a process essential for chromosome congression. Small molecule inhibitors of CENP-E, which can disrupt the mitosis during cell division, are reported as anticancer drugs. For instance, GSK923295 is a CENP-E inhibitor currently undergoing clinical trials, which works by locking CENP-E bound to microtubules, preventing the release of inorganic phosphate during its ATPase cycle. Tamaoki *et al.* developed a photoswitchable inhibitor based on azopyrazole that targets CENP-E and showed reversible control of CENP-E activity in cancer cells by UV light exposure with a 10-fold difference in inhibition activity between *trans* and *cis* isomers (Fig. 6b).^97^ This study highlights the potential of a photochemical approach for precisely controlling mitotic interference and uncovering specific molecular functions in dynamic cellular processes. By slightly changing the molecular structure, they recently showed that the photocontrol of CENP-E inhibition is possible by using only visible light making them better suitable for clinical applications.^98^ Another category of photoswitchable kinase inhibitors reported by the same group includes rho-associated coiled-coil-containing protein kinase (ROCK) that plays a crucial role in regulating the actomyosin essential for cell movement and shape.^99^ The inhibitor design was based on a phenylazothiazole scaffold, which allows them to use visible light for switching inhibitory behavior between its active (*trans*) and inactive (*cis*) forms in living cells. The photocontrol of these kinase inhibitors is particularly useful for studying cellular processes and may lead to the development of new therapeutic strategies.

### 4.5 Histone deacetylase inhibitor

**Fig. 7:**
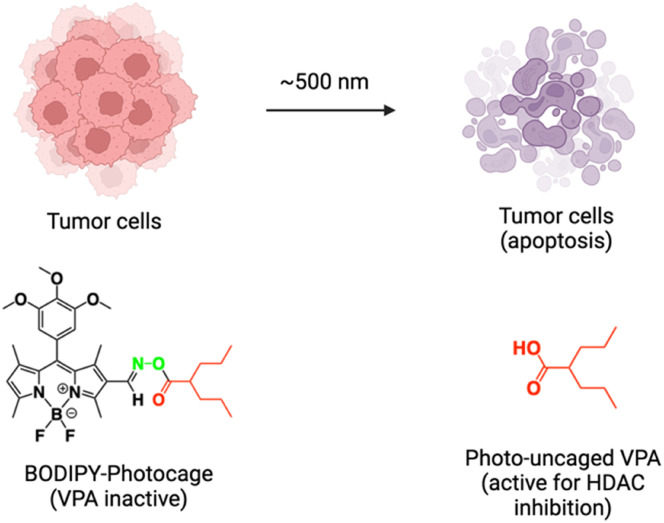
Scheme illustrating the visible light-triggered uncaging of the BODIPY-photocage conjugated to VPA along with photocontrol of apoptosis in tumor cells.

Zhang *et al.* demonstrated photo-uncaging by longer wavelength green light irradiation by introducing a BODIPY group to the valproic acid (VPA) drug *via* a light-cleavable N–O linkage.^100^ VPA has shown potential as an anticancer agent due to its ability to inhibit histone deacetylase (HDAC) and induce apoptosis; however, its direct administration is associated with severe side effects.^101^ In their case, the caged VPA was efficiently taken up by tumor cells as observed from microscopy images and remained inactive in the dark. Upon exposure to green light (∼500 nm), the active form of VPA was released within tumor cells and inhibited HDAC leading to apoptosis and cell death (Fig. 7). Importantly, the IC50 value for VPA inhibition in HeLa cells obtained by the photo-uncaging method was about 100 times lower than the IC_50_ value of VPA alone without any photocontrol, demonstrating the therapeutic benefit of photo-uncaging in drug action.

### 4.6 TGF-β inhibitor

**Fig. 8:**
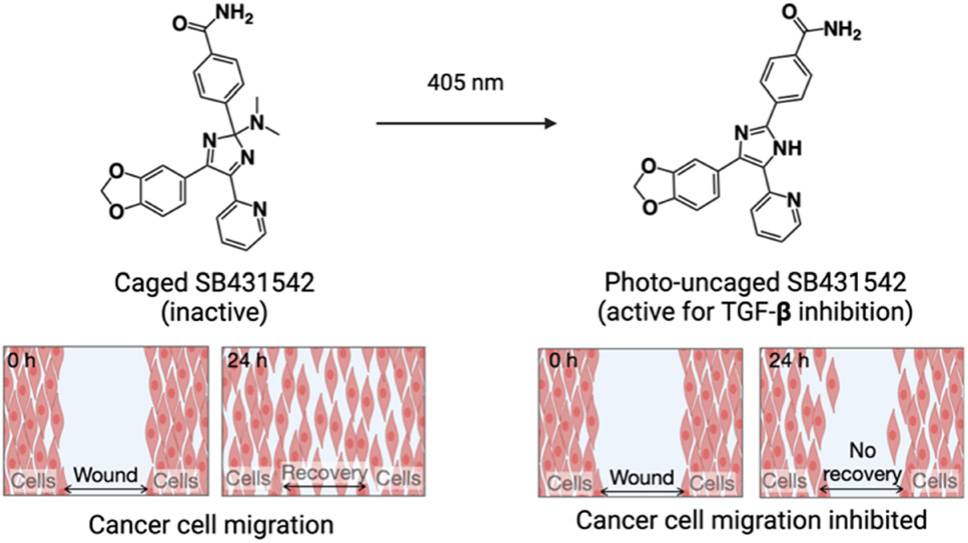
Scheme illustrating the visible light-triggered uncaging of caged SB431542 along with photocontrol of cell migration in the wound healing assay.

Tamaoki *et al.* developed a versatile photocaging strategy targeting the triarylimidazole moiety, an essential structural component of many small-molecule kinase inhibitors used in anticancer therapies.^54,102^ This method involved modifying SB431542, a well-established triarylimidazole-based inhibitor of TGF-β receptor 1, which effectively suppresses tumor-promoting processes such as cell migration and invasion. By introducing a dimethylamino group at the carbon atom of the imidazole ring, the originally planar structure of SB431542 was transformed into a tetrahedral configuration, resulting in significant differences in receptor affinities between the caged and uncaged forms. Photo-uncaging was achieved using 405 nm light, enabling precise spatiotemporal control over drug activation in a cellular environment. The utility of this method was demonstrated in a wound-healing assay, where light-mediated activation of the caged compound selectively inhibited wound recovery in illuminated regions by releasing the active inhibitor, while unilluminated regions exhibited normal wound healing due to the presence of the inactive, caged form (Fig. 8). These findings highlight the potential of this approach for achieving targeted regulation of pharmacological activity in complex biological systems.

### 4.7 Photoactivable neurons

Light controlled methods for site-selective drug action in the brain are very limited.^103^ Banghart *et al.* developed photoactivatable derivatives of oxymorphone and naloxone, an agonist and antagonist, respectively, of the mu-opioid receptor (MOR).^104^ They incorporated dimethoxynitrophenethyl (DMNPE) as the caging group, administered to mice *via* intravenous injection and then activated locally in the brain using UV light through an optical fiber. They found that photoactivation resulted in significant MOR occupancy only at the site of light irradiation. The use of DMNPE caging groups ensured compatibility with green and red fluorescent probes commonly used in *in vivo* imaging, as DMNPE does not absorb visible light. They also showed the simultaneous optical recording and drug photoactivation through the same optical fiber, enhancing the utility of photopharmacology for studying neural behavior.

## 5. Challenges in the field of photomedicine

As a conceptual expansion in the field of photomedicine, the current focus is photopharmacology that includes PDT. Unlike phototherapy that uses UV light as a source of therapeutic effect, photopharmacology works based on the interaction with light and the photoactive molecular drug. Hence the challenges associated with photopharmacology include the localized delivery of both the pro-drug and light at the target site, concerns related to the adverse effect of both the pro-drug and light, and undesired photochemical reactions in the bioenvironment. Accordingly, to achieve control over drug activity through photopharmacology, a detailed understanding of various factors, particularly the structural design of photopharmacological agents is necessary.^75^ Upon light-induced activation, such as uncaging or isomerization, these agents must undergo significant structural changes, yielding a marked difference in receptor affinity between their inactive and active states. This change in affinity is essential for precise control, as it ensures that only the active form of the drug binds with its target receptor. Some of the studies reported so far in the field of photopharmacology attempted to understand the basic difference in biological response with or without light in cellular level experiments and exceptionally in animal models.^75^ Hence the nature of light (UV or visible) was not a priority to consider but rather the incorporation of the photo-active segment in the molecular design. However, as the field advances, the choice of light also started to attract much attention and many new molecular designs that can show a difference in biological response by visible light irradiation were reported. For instance, by incorporating the bulky aromatic substituents in the photocaging compounds, it is now possible to uncage the compound for target biological response by longer visible and NIR wavelengths of light.^50–53^ In the case of photoswitches, substitutions at the phenyl segment by halogens or electron donating groups made the switching light shift to longer wavelengths, but with a compromise in the thermal stability of the *cis* isomer.^105^ Another aspect is the mode of light delivery to ensure the light reachability to the target site. External delivery of light is the most common in terms of accessibility; however penetration depth through the skin layers is still very limited. New photonic technologies such as optical fibers can overcome this challenge and deliver light into deep tissues with spatial precision.^106^ Recent reports showed effective methods of photomedicine using an optical fiber.^107^ Importantly, fiber-optic PDT endoscopy is now available in clinical practice demonstrating the potential of optical fibers in photomedicine.^108^ Photopharmacological agents, especially photoswitches and photocaging groups, typically rely on single-photon excitation (1PE), where all molecules within the irradiated area absorb light and undergo isomerization or uncaging, potentially limiting spatial control. Indirect isomerisation with low-energy photons is an alternative method for photoisomerisation by longer wavelengths.^109^ This includes two-photon excitation, triplet sensitization, photon upconversion, photoinduced electron transfer, and indirect thermal methods.^110,111^ Gorostiza *et al.* showed the use of two-photon excitation for the photoactivation of neurons and astrocytes with subcellular resolution in azobenzene-based protein switches.^112^ They recently demonstrated the regulation of neuronal activity in zebrafish larvae using three-photon excitation (3PE) of a photoswitchable muscarinic agonist at a very low concentration of 50 pM.^113^ Importantly, this was achieved using mid-infrared light at 1560 nm, the longest wavelength ever reported for photoswitching. However, light is also strongly absorbed by water at this wavelength and hence may not be the ideal spectral range for clinical application of 3PE.

Incomplete isomerization is another significant limitation in the use of photoswitches for photopharmacology, as it leads to an incomplete transition between the active and inactive forms of the drug. At photostationary states, both isomeric forms (*cis* and *trans*) coexist, resulting in only partial activation or deactivation of the photoswitchable molecule. This incomplete switching can reduce the contrast in biological activity between the two states, which can decrease the efficacy of the treatment. Another important consideration is the stability of photoactive compounds under light exposure, especially in biological environments rich in reducing agents such as glutathione (GSH).^114^ For instance, GSH can react with an azobenzene chromophore, one of the most investigated motifs for photopharmacology studies, converting it to non-photoswitchable hydrazine derivatives. Another challenge is the diffusion of active species generated by light irradiation that may rapidly diffuse through the cellular environment, spreading beyond the target area and reducing spatial resolution. The rate of diffusion, relative to the timescale of the biological process and the lifetime of the active species, determines the specificity of the drug's action. Furthermore, certain azo compounds have been associated with mutagenic and carcinogenic risks.^115^

## 6. Conclusions

In this review we have discussed the brief history of light therapy and photomedicine, and the emergence of a new field nowadays known as photopharmacology. We focused the discussion based on the mode of actions in photopharmacology such as photodynamic, photo-uncaging and photoswitchable therapeutical methods, along with a few representative examples that showed a difference in biological response under dark and light conditions. We also briefly discussed the “biological window” of light, which is most important for achieving therapeutic benefits. As the recent focus in the field was about photoswitch-based photopharmacology, we included a section about its design principles especially for developing longer wavelength responsive systems. Although photomedicine has undergone significant advancements over the years, only light therapy and PDT have progressed to clinical applications and others are still in the level of fundamental research. At this end, we have briefly mentioned the challenges including light properties (1D and multi-photon), molecular design (photo-uncaging and photoswitching) and clinical translations. Overcoming these challenges could enable photopharmacology to fulfill its potential in precision medicine, offering new approaches for treating diseases effectively without any troublesome side effects. Photopharmacology goes beyond simply combining light with pharmacologically active drugs; it involves modifying drugs to be photo-activatable. As a result, it will likely take time for these innovations to reach the public market. We foresee the bright future of photomedicine in cancer chemotherapy with enhanced antitumor effect precisely at the target site.^116^

## Conflicts of interest

There are no conflicts to declare.

## Data Availability

No primary research results, software or code have been included and no new data were generated or analysed as part of this review. The discussed articles were cited properly in the review manuscript.
